# Dynamics and Malleability of Plant DNA Methylation During Abiotic Stresses

**DOI:** 10.3390/epigenomes9030031

**Published:** 2025-08-29

**Authors:** Niraj Lodhi, Rakesh Srivastava

**Affiliations:** 1 Department of Pathology and Genomic Medicine, Thomas Jefferson University, Philadelphia, PA 19107, USA; 2 Research and Development, Helix Biosciences, New Delhi 110028, Delhi, India

**Keywords:** gene regulation, epigenetics, DNA methylation, stress, drought, salinity, heat, cold, heavy metal, epigenetic memory

## Abstract

Epigenetic regulation, particularly DNA methylation, plays a crucial role in plant adaptation to environmental stresses by modulating gene expression without altering the underlying DNA sequence. In response to major abiotic stresses such as salinity, drought, heat, cold, and heavy metal toxicity, plants undergo dynamic changes in DNA methylation patterns. These modifications are orchestrated by DNA methyltransferases and demethylases with variations depending on plant species, genetic background, and ontogenic phase. DNA methylation affects the expression of key genes involved in cellular, physiological, and metabolic processes essential for stress tolerance. Furthermore, it contributes to the establishment of stress memory, which can be transmitted across generations, thereby enhancing long-term plant resilience. The interaction of DNA methylation with other epigenetic mechanisms, including histone modifications, small RNAs, and chromatin remodeling, adds layers of regulatory complexity. Recent discoveries concerning N6-methyladenine have opened new avenues for understanding the epigenetic landscape in plant responses to abiotic stress. Overall, this review addresses the central role of DNA methylation in regulating plant stress responses and emphasizes its potential for application in crop improvement through epigenetic and advanced biotechnological approaches.

## 1. Introduction

Plants, being sessile, depend on advanced regulatory mechanisms to cope with environmental and biotic stresses, with epigenetic regulation playing a key role. This involves DNA methylation, histone modifications, chromatin remodeling and non-coding RNAs, which together shape the dynamic epigenome. Epigenetic mechanisms govern key developmental processes like germination, flowering, and organ formation and enable phenotypic plasticity and adaptive memory to environmental cues such as drought, heat, and pathogens [1,2,3,4,5,6].

Abiotic stresses such as drought, salinity, extreme temperatures, cold, and heavy metal toxicity disrupt photosynthesis, nutrient uptake, and reproduction, causing oxidative damage, water imbalance, and metabolic dysfunction that reduce biomass, yield, and crop quality [4,7,8,9,10]. In response to these stresses, plants regulate gene expression through transcriptional control and epigenetic modifications [1,5,11,12,13]. Promoters, which recruit RNA polymerase and transcription factors, are key regulatory regions whose epigenetic modification status influences gene activity [14,15]. These modifications including DNA methylation and histone modifications modulate gene expression without altering DNA sequences and play essential roles in stress tolerance and defense [12,16,17]. Some epigenetic changes are reversible, while others are heritable, contributing to stress memory and transgenerational epigenetic adaptation [18,19]. Abiotic stresses often alter DNA methylation patterns, influencing the expression of stress-responsive genes [20,21]. Depending on its location, DNA methylation can repress or activate genes. Under stress, plants exhibit dynamic methylation changes that silence growth-related genes and activate defense genes, adjusting metabolism and physiology to survive adverse conditions. These epigenetic adjustments sometimes enable the inheritance of stress memory across generations [20,22]. This review aims to explore the impact of major abiotic stresses, including salinity, drought, heat, cold, and heavy metal toxicity, on DNA methylation in plants, and to examine how these epigenetic changes influence plant biological responses and metabolic pathways.

## 2. Plant DNA Methyltransferases and Demethylases: An Insight

In plants, DNA methylation predominantly involves the addition of a methyl group to either the fifth carbon of cytosine, forming 5-methylcytosine (5mC), or to the sixth nitrogen of adenine, producing N6-methyladenine (6mA). These two epigenetic marks play crucial roles in regulating gene expression, maintaining genome integrity, and facilitating plant responses to environmental stresses. Cytosine methylation (5mC) occurs in three distinct sequence contexts: CG, CHG, and CHH (where H represents A, T, or C). Each of these contexts is regulated by specific DNA methyltransferases and is differentially distributed across genomic regions [3,17,22].

**Figure 1:**
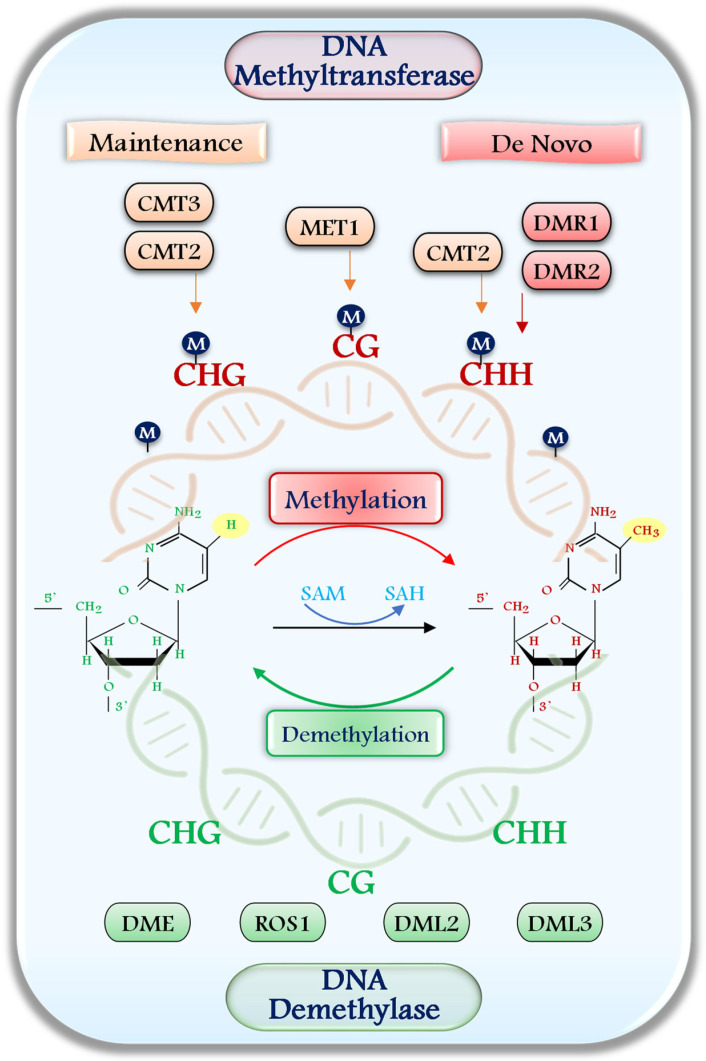
Representation of DNA methylation at 5-methylcytosine (5mC) by plant DNA methyltransferases and demethylation by DNA demethylases is well characterized in the model plant *Arabidopsis thaliana*. DNA methylation is catalyzed by DNA methyltransferases using S-adenosyl methionine (SAM) as a methyl donor, which results in the addition of a methyl group to cytosine bases, forming 5-methylcytosine and releasing S-adenosyl homocysteine (SAH). Methylation at CG sites is primarily maintained by METHYLTRANSFERASE 1 (MET1), whereas CHG methylation is maintained by CHROMOMETHYLASE 3 (CMT3) and CHROMOMETHYLASE 2 (CMT2). CHH methylation, which is asymmetrical, is guided by both CMT2 and de novo methyltransferases such as DOMAINS REARRANGED METHYLTRANSFERASE 1 (DRM1) and DOMAINS REARRANGED METHYLTRANSFERASE 2 (DRM2). In contrast, DNA demethylation involves the removal of methyl groups and is carried out by enzymes including REPRESSOR OF SILENCING 1 (ROS1), DEMETER (DME), DEMETER-LIKE 2 (DML2), and DEMETER-LIKE 3 (DML3). This active DNA demethylation process plays a crucial role in reversing epigenetic marks and fine-tuning gene expression in response to developmental signals and environmental stimuli.

Plant DNA methyltransferases (DNMTs) are functionally classified into de novo methyltransferases and maintenance methyltransferases (Figure 1), each playing distinct but complementary roles in the establishment and preservation of DNA methylation patterns [21,23]. De novo methyltransferases are responsible for establishing new methylation marks on previously unmethylated DNA and are crucial during developmental transitions and environmental responses. In plants, this function is primarily executed by DOMAINS REARRANGED METHYLTRANSFERASE 1 and 2 (DRM1 and DRM2), which are homologous to the DNMT3 family in animals [2,3,24,25]. Among them, DRM2 is the major catalytically active enzyme, while DRM1 is typically expressed at low levels and is considered catalytically inactive or minimally active under most conditions [2,26]. DRM1/2-mediated methylation is directed by small interfering RNAs (siRNAs) via the RNA-directed DNA methylation (RdDM) pathway [23,27]. This epigenetic mechanism involves the coordinated action of siRNAs, scaffold RNAs transcribed by RNA polymerase V, and various effector proteins that recruit the methyltransferase complex to target loci. Key to the RNA-directed DNA methylation (RdDM) pathway in plants are two plant-specific RNA polymerases, Pol IV and Pol V, both derived from RNA polymerase II. Pol IV initiates siRNA biogenesis by generating transcripts that are converted into 24-nt siRNAs, while Pol V produces scaffold RNAs at target loci [28]. RdDM predominantly catalyzes CHH context methylation in euchromatic regions and at the boundaries of transposable elements, contributing to transcriptional silencing and genome stability [2,29,30]. Importantly, RdDM also plays a role in transgenerational epigenetic inheritance, supported by feedback mechanisms that reinforce methylation at specific loci across generations [31]. CLSY and SHH1 play crucial roles by guiding the methylation machinery to targeted genomic regions, ensuring the precise establishment and long-term maintenance of methylation patterns [32,33]. Maintenance methyltransferases, in contrast, act during DNA replication to preserve existing methylation patterns by recognizing hemimethylated DNA and restoring symmetric methylation [3]. In plants, METHYLTRANSFERASE 1 (MET1) serves as the primary enzyme for CG methylation maintenance, functioning analogously to DNMT1 in mammals [34]. Non-CG methylation is maintained by the CHROMOMETHYLASE (CMT) family. CMT3 mainly adds methylation to CHG sites, while CMT2 works on CHH sites and some CHG sites, especially in heterochromatin regions. These regions have a special histone mark called H3K9me2, which helps CMT2 and CMT3 bind to the DNA. This interaction guides them to add methylation at the right spots, helping to keep the structure and function of heterochromatin [23,30,35]. These maintenance enzymes are guided by the chromatin landscape, ensuring that epigenetic silencing is retained in repetitive DNA regions and transposable elements. Together, de novo and maintenance methyltransferases orchestrate the dynamic yet heritable DNA methylation landscape in plants, enabling both stable gene repression and flexible responses to environmental and developmental cues.

The regulation of gene expression through dynamic DNA methylation requires not only the addition but also the removal of methyl groups from 5mC (Figure 1). In plants, DNA demethylation occurs via two principal pathways: passive and active demethylation [2,23]. Passive DNA demethylation takes place during DNA replication when DNA methyltransferases are either inactive, absent, or their activity is limited due to a shortage of methyl donors such as SAM. Under these conditions, the newly synthesized DNA strands remain unmethylated, resulting in hemimethylated or demethylated daughter strands, leading to a progressive dilution of 5mC across successive cell divisions. This mechanism is particularly relevant under stress conditions or developmental transitions that modulate methyltransferase expression or activity. Active DNA demethylation in plants is mediated by DNA demethylases (dMTase) through a base-excision repair (BER) pathway. In this pathway, four bifunctional 5mC DNA glycosylases, DEMETER (DME), REPRESSOR OF SILENCING 1 (ROS1), and their homologs DEMETER-LIKE PROTEIN 2 (DML2) and DML3, are involved in actively removing 5-mC from various sequence contexts [21,23]. These glycosylases excise 5mC and initiate the BER mechanism, which plays a crucial role in maintaining the dynamic regulation of DNA methylation in plants [3,20,21]. DML2 and DML3, members of the ROS1/DME family, help remove DNA methylation in different plant tissues. Like ROS1 and DME, they play key roles in active demethylation during various stages of plant development. These enzymes mainly target promoter regions, transposable elements, and imprinted genes, helping to maintain genome stability and proper gene expression [22,36,37]. DML2 and DML3, as members of the same family, contribute to DNA demethylation in a tissue-specific manner and during different developmental stages, highlighting the functional redundancy and specialization within this enzyme family [21,23]. For example, ROS1, DML2, and DML3 are ubiquitously active in vegetative tissues, but plants lacking all three mutants showed DNA hypermethylation at about 9000 loci, nearly twice the 5000 loci affected by ROS1 alone, suggesting each has a specialized role [38,39]. Together, these pathways ensure the fine-tuned and reversible control of DNA methylation, essential for developmental plasticity and environmental adaptability in plants.

**Figure 2:**
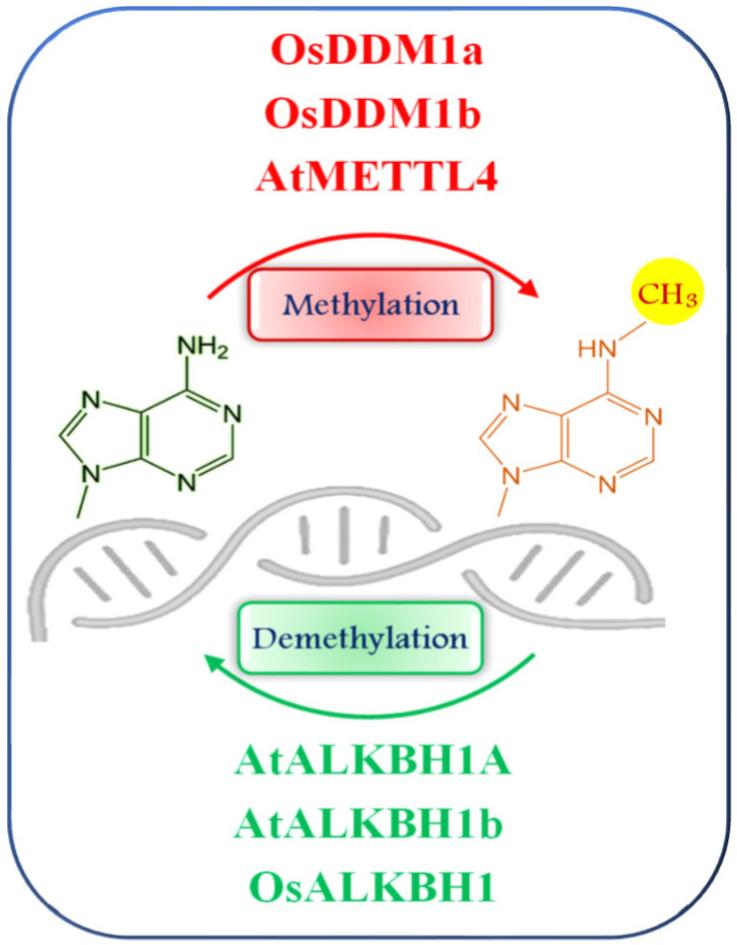
DNA 6mA modification is catalyzed by methyltransferases like AtMETTL4 in Arabidopsis and OsDDM1a/b in rice, while demethylation is mediated by demethylases including AtALKBH1a/b in Arabidopsis and OsALKBH1 in rice.

N6-methyladenine (6mA) DNA methylation has recently emerged as a novel epigenetic modification in eukaryotes, distinct from the well-characterized 5mC (Figure 2). In plants, 6mA is increasingly recognized for its role in gene regulation, development, and stress responses. Unlike 5mC, which is generally associated with transcriptional repression, 6mA within gene bodies is positively correlated with gene expression. This association has been demonstrated in both *Arabidopsis thaliana* and *Oryza sativa*, where 6mA enrichment within coding regions is linked to active transcription [40,41], However, promoter-localized 6mA appears to play a repressive role, particularly in rice, where it is associated with gene silencing [42]. Enzymatic machinery for 6mA regulation has been partially characterized. In mammals, 6mA is deposited by N6MT1 and removed by the demethylase ALKBH1 [43,44]. In *Arabidopsis*, METTL4 has been identified as the methyltransferase responsible for catalyzing 6mA, while AtALKBH1A and AtALKBH1D act as 6mA demethylases [45,46]. In rice, 6mA modification requires the chromatin remodelers OsDDM1a and OsDDM1b, and demethylation is mediated by OsALKBH1 [41]. Despite its functional significance, 6mA is relatively rare compared to 5mC in plant genomes. Genome-wide mapping indicates that 6mA is broadly distributed across plant genomes and conserved in both plants and mammals. In *Arabidopsis*, the global 6mA content ranges from 0.006% to 0.138%, whereas in rice seedlings it is slightly higher, ranging from 0.15% to 0.55% of total adenines [40,41,42]. It is enriched at specific sequence motifs, notably ANYGA and GAGG, suggesting a degree of sequence specificity [40]. In rice, approximately 20% of protein-coding genes and 14% of transposable elements harbor 6mA modifications. In *Arabidopsis*, about 32% of 6mA sites are located within gene bodies, reinforcing its role in transcriptional activation [40,42]. Collectively, these findings highlight 6mA as a versatile and evolutionarily conserved epigenetic mark that adds complexity to plant gene regulation and offers promising avenues for exploring stress adaptation and developmental control.

## 3. DNA Methylation in Abiotic Stress

Abiotic stresses alter DNA methylation landscapes, causing both hypermethylation and hypomethylation that affect gene expression and promote plant adaptation. These methylation changes often modulate gene expression, transposable element activity, and stress memory, thereby enhancing plant adaptation and plasticity; such epigenetic changes can result in heritable epialleles that influence long-term stress resilience [4,7,47,48]. Epialleles are heritable gene variants defined by epigenetic modifications, particularly DNA methylation, rather than DNA sequence changes. Methylation at promoters or regulatory regions can switch genes on or off, generating phenotypic diversity. Classic examples of epialleles include *FWA* and *FLC* in *Arabidopsis* (flowering time), *GhCOL2* in cotton (photoperiodic flowering), *CNR* in tomato (non-ripening), and *Lcyc* in toadflax (floral symmetry) [49,50]. These cases illustrate how methylation-driven epialleles influence development and stress adaptation, while stress can generate novel epialleles that enhance plasticity without DNA changes [51,52].

DNA methylation responses to stress are highly variable and shaped by multiple interacting factors, including sequence context (CG, CHG, CHH), regulatory targets, species-specific genomic features, tissue identity, and the timing of stress exposure. Although CHH methylation changes are frequently reported, stress can alter methylation across all sequence contexts. These modifications could occur in both genes and transposable elements, with their functional impact depending on genomic location and regulatory relevance. Furthermore, Species-level differences, such as genome size, transposable element abundance, and reproductive strategies, add further complexity, while tissue-specific responses reflect the distinct functional requirements of different cell types. Importantly, methylation changes can be transient or stable, often occurring before, during, or after gene expression shifts, underscoring their dynamic nature. Collectively, these factors explain there is no universal pattern of hyper- or hypomethylation emerges under stress. Instead, stress adaptation relies on a flexible, con-text-dependent epigenetic regulation system that enables organisms to fine-tune their responses to diverse environmental challenges [53,54,55].

### 3.1. DNA Methylation During Salt Stress

Soil salinity disrupts plant productivity, posing a serious risk to food systems and agricultural sustainability. High salinity disrupts osmotic and ionic balance and impairs key physiological processes such as protein synthesis, photosynthesis, and metabolism. Salt stress triggers dynamic and often reversible alterations in DNA methylation patterns across different plant species. These changes can be tissue-specific, genotype-dependent, gene-specific, and developmentally regulated, playing a pivotal role in modulating gene expression involved in ion homeostasis, antioxidant defense, and hormonal signaling.

To better understand the mechanistic relevance of DNA methylation under salt stress, species-specific investigations have revealed a wide array of methylation changes, often linked with differential stress tolerance. For instance, DNA methylation changes in salt-stressed wheat are genotype- and tissue-specific and are linked to downregulation of salt-responsive high-affinity potassium transporter (*HKT*) genes, which help maintain sodium and potassium ion balance under salt stress. In the tolerant variety Kharchia-65, increased methylation in the 5′ proximal coding regions of *TaHKT2;1* and *TaHKT2;3* reduces their expression in roots and shoots, thereby enhancing salt tolerance. However, *TaHKT1;4* is downregulated in roots independently of methylation [56]. In *Medicago truncatula*, salt stress increased DNA methylation at CpG sites (5′-CCGG-3′) and genome-wide, accompanied by a 5- to 10-fold upregulation of two DNA methyltransferase homologs under 400 mM NaCl. This response is likely driven by methyltransferase activation, as treatment with the DNA methylation inhibitor 5-azacytidine reduced salt tolerance. An ELISA-based assay further revealed a 26% increase in DNA methylation in alfalfa roots exposed to 20 dS/m salinity, indicating global DNA methylation changes under salt stress [57]. Kenaf, a salt-tolerant industrial fiber crop, shows decline in DNA methylation in response to salt stress while co-treatment with 5-azacytidine significantly enhanced the salt tolerance of seedlings. This treatment boosts biomass and antioxidant enzyme activities while reducing oxidative damage markers. MSAP and qRT-PCR analyses showed reduced methylation and altered expression of stress-responsive genes (*ADH*, *GLR3.4*, *GBPa1*, *L-AAO*, *NAC67*, and *TLC34*), suggesting that DNA demethylation improves kenaf’s resilience to salt stress [58]. Similarly, in faba bean, application of 5-azacytidine reduced methylation and enhanced salt tolerance. Salinity stress in faba bean induces DNA methylation, reducing germination, increasing chromosomal abnormalities, and upregulating DNA methyltransferase genes (*MT1* and *MT2*), while downregulating antioxidant and stress-related genes (*CAT*, *Cu/Zn-SOD*, *GR*, *GPRP*, and *HSP-17.9*). Treatments with 5-azacitidine improved physiological traits, lowered DNA methylation, and restored gene expression, suggesting these treatments can mitigate salinity stress [59]. In Arabidopsis, salt stress (20 and 75 mM NaCl) caused a significant decrease in CG methylation in the coding region of homeobox gene *Glabra-2*, a key role in root epidermal cell differentiation. This demethylation was more pronounced under severe stress (33% vs. 23% in mild stress) and was associated with an increased number of root hair-forming trichoblasts [60].

Comparative analyses across plant species reveal diverse epigenetic mechanisms contributing to salt stress tolerance. In pigeonpea (*Cajanus cajan* L.), salt tolerance was compared between tolerant (ICP7) and sensitive (ICP1071) genotypes. The tolerant ICP7 genotype exhibited better stress protection, elevated gene activity, and a more substantial reduction in DNA methylation. Notably, salt stress triggered specific CHH methylation in the coding region of *CcCDR* (cold and drought-regulatory protein) gene in ICP7, suggesting that targeted methylation events may contribute to enhanced salt tolerance [61]. Similarly, in wheat, lines introgressed with *Aegilops cylindrica* (BC4F2) demonstrated enhanced salinity tolerance, which was associated with improved ion balance, elevated antioxidant levels, and upregulation of the *NHX1* and *SOS1* genes. Salinity stress induced a more pronounced reduction in 5mC levels in the tolerant lines, especially in leaf tissues, pointing to successful epigenetic introgression and tissue-specific methylation responses [62]. In rice, seed germination under salt stress was compared between salt-tolerant FL478 and salt-sensitive IR29 genotypes. FL478 exhibited higher germination rates under stress, whereas IR29 displayed increased expression of *GD1* (germination defective 1), a gene influencing seed germination, along with stronger salt-responsive gene expression changes overall. Both genotypes showed increased CHH methylation in transposable elements regions under salt stress. In IR29, these methylation changes were linked to stress and germination-related genes, highlighting the interplay between genetic and epigenetic factors in rice salt tolerance during early developmental stages [63]. Supporting this, genome-wide analysis in salt-tolerant rice identified differentially methylated regions (DMRs) that undergo demethylation in response to salinity stress. These DMRs were associated with altered expression of adjacent genes, suggesting that they may influence chromatin structure and activate stress-responsive pathways. The identification of such regulatory regions provides new molecular targets for improving plant salt tolerance through epigenetic modification [64]. Expanding the analysis to sugar beet, salt stress-induced DNA methylation changes were found to differ between two subspecies. DNA methylation in specific CpG islands responded in a gene- and species-specific manner. *Beta vulgaris* (sugar beet) exhibited predominant promoter hypomethylation, whereas the salt-tolerant *B. maritima* showed more frequent hypermethylation. These contrasting epigenetic strategies point to the role of DNA methylation in shaping adaptive responses to salinity. Furthermore, inverse correlations between methylation levels and expression of stress-responsive genes emphasize DNA methylation’s role in regulating gene activity under stress, offering promising targets for future breeding strategies [65].

In parallel with these phenotypic and methylomic shifts, salt stress also exerts regulatory control over the expression of DNA methylation machinery, particularly DNA methyltransferases and demethylases, indicating a coordinated epigenetic response. Salt stress also alters the expression of DNA methyltransferases (MTases) and demethylases (DMLs), often in a tissue- and genotype-specific manner. In cotton, the expression of DNA methylation genes, especially C-5 cytosine-specific DNA MTases, was modulated in different tissues of salt-tolerant and sensitive genotypes under NaCl stress [66]. Notably, *GhDMT6* was highly upregulated, and its silencing via VIGS improved seedling stress resistance, highlighting its regulatory role in cotton stress responses [66]. Similarly, in pear, the *MET*, *CMT*, *DRM*, and *DNMT2* gene families showed differential expression in roots and leaves in response to NaCl [67]. Most MTase genes were suggested increase in roots, particularly *PbeMET1a*, *PbeCMT3*, and *PbeDRM1–3*, while stems generally showed downregulation. In leaves, a few genes (*PbeMET1a/b*, *PbeDRM2*, *PbeDnmt2b*) were suggested increase, with others repressed. Salt stress also increased DNA methylation in the promoters of *PbeNHX2.1*, *PbeCBL2* (CpG), and *PbeAKT2* (CHG/CHH), correlating with gene repression and indicating a regulatory role in stress response [67]. Fan et al. (2020) identified 22 DNA methylase and 6 demethylase genes in rapeseed. Under salt stress, most *BnaMETs* were correlated to decrease in expression, *BnaDNMT2s* were suggested increase, and *BnaDRMs* showed mixed responses. Salt stress also modulated the expression of demethylase genes such as *ROS1* and *DML*, indicating a dynamic epigenetic response to salinity in the polyploid plant rapeseed [68]. Salinity significantly altered the expression of DNA methylation-related genes in *Bruguiera gymnorhiza*. Under natural and control saline conditions, *BgCMT2* and *BgCMT3* were suggested increase and de novo methyltransferase *BgDRM2* (involved in RdDM) was also suggested upregulated, while the demethylase *BgDME2* was found downregulated [69]. In eggplant, under salt stress, the hybrid ‘Nite Lady’ showed downregulation of *SmelCMT3a/b* and upregulation of *SmelDRM2/3* and several demethylases, indicating dynamic DNA methylation changes [70]. Dynamic expression of demethylase genes also supports salt responses in foxtail millet. Under NaCl stress, most DNA demethylase genes increased in expression. *SiDML5*, *SiDML3a*, and *SiDML3b* showed steady increases, while *SiDML4*, *SiROS1a*, *SiROS1b*, and *SiROS1d* peaked early and declined thereafter, indicating temporal regulation of methylation during stress [71].

Changes in the expression of DNA methylation enzymes are closely linked to the methylation status of stress-regulatory genes, influencing their expression and contributing to salt stress tolerance. For example, Arabidopsis demethylase *AtROS1* overexpression in tobacco led to enhanced salt tolerance, reduced H_2_O_2_ levels, and increased flavonoid accumulation by regulating the methylation status of key genes (*CHS*, *CHI*, *F3H*, *FLS*, *DFR*, *ANS*, *GST*, *APx*, *GPx,* and *GR*) in the flavonoid biosynthesis and antioxidant defense pathway. The elevated expression of these stress-responsive genes in transgenics correlates with *AtROS1*-mediated demethylase activity, highlighting the critical role of DNA methylation dynamics in modulating plant defense and salt tolerance [72]. Additionally, a study on *Arabidopsis* suspension cells revealed that DNA methylation plays a protective role against salt-induced mutagenesis. Methylation-deficient mutants (*ddc* and *nrpe1*) showed greater salt adaptability but also accumulated more mutations under salt stress compared to wild-type cells. These mutations were not enriched in stress-responsive genes, suggesting that DNA methylation contributes to genomic stability during stress adaptation [73].

A pivotal pathway mediating methylation in response to salt stress is the RdDM mechanism, which governs CHH methylation and plays a critical role in regulating gene expression under salinity. A recent study highlights the role of RdDM pathway in maize salt stress tolerance. ZmKTF1, a KOW domain-containing transcription factor essential for RdDM, is key to this response. Mutations in *ZmKTF1* reduce CHH methylation and disrupt the expression of oxidoreductase-related genes (*RBOH*, respiratory burst oxidase homolog related genes), resulting in elevated ROS levels under salt stress. These findings establish ZmKTF1 as a crucial epigenetic regulator of salt tolerance and a promising target for maize improvement [74]. In Arabidopsis, the splicing factor *RDM16* enhances salt stress tolerance by modulating ABA sensitivity and regulating stress-responsive genes. As a key component of the RdDM pathway, RDM16 influences methylation at transposable elements and gene regions. Its mutation reduces DNA methylation and Pol V transcripts, without affecting small RNAs, leading to increased sensitivity to salt and ABA stress [75]. Another study in *Arabidopsis thaliana* revealed that epigenetic regulation of *AtHKT1*, a key salt tolerance gene, relies on both a distal enhancer and RdDM-mediated CHG and CHH methylation in an upstream region. This methylation ensures tissue-specific *AtHKT1* expression in leaves and roots. Disruption in mutants (*rdr2*, *met1-3*) or lines with altered enhancer positioning caused reversed expression, Na^+^ overaccumulation in leaves, and increased salt sensitivity, highlighting epigenetic control’s vital role in Na^+^ homeostasis and salt stress resilience [76]. In *Bruguiera gymnorhiza*, salt stress triggers genome-wide non-CG hypermethylation in transposable elements and gene regions via RdDM and CMT pathways. This is accompanied by upregulation of *DRM* and *CMT* methyltransferases and downregulation of demethylases, suggesting an epigenetic mechanism for TE silencing and salinity adaptation [69]. Similarly, in chickpea, salt stress leads to distinct DNA methylation patterns that regulate gene expression. The tolerant genotype shows increased CG methylation in genes and CHH methylation in transposable elements, mediated by small RNAs, indicating a coordinated epigenetic mechanism underlying salt tolerance [77].

In addition, there is a strong interaction between phytohormones and DNA methylation under salt stress, emphasizing a multi-layered regulatory network integrating epigenetic and hormonal signaling. In sorghum, genome-wide DNA methylation analysis showed that treatment with gibberellic acid (GA_3_) changed DNA methylation in 18,032 regions affecting 6093 genes. Among these, 337 genes were enriched in stress-related pathways like phenylpropanoid biosynthesis and amino acid metabolism. Key genes, including cytochrome P450 84A1, aldehyde dehydrogenase family members C4 and 7A1, peroxidases 70, 2, and 4, aminotransferase TAT2, aromatic-L-amino-acid decarboxylase, and polyamine oxidase, showed significant methylation changes and were validated, supporting GA_3_’s role in regulating salt stress responses through DNA methylation and modulated the expression of genes involved in phenylpropanoid and amino acid metabolism [78]. Strigolactones (SLs) are multifunctional plant hormones that regulate development and architecture while also playing key roles in plant adaptation to abiotic stresses [79]. In tomato, transcriptome and genome-wide methylation analyses showed that treatment with SLs and the DNA methylation inhibitor 5-azacytidine relieve salt stress in tomato led to a reduction in CHG methylation and by enhancing growth and metabolite production. SLs activate key stress-response pathways, especially phosphatidylinositol signaling (*PIP2*, *DAG*, *IP3*, *PA*), and upregulating related genes elated genes (e.g., *SlPLC2*, *SlPLD-Z*, *SlDGK1*) by reducing CHG methylation, suggesting SL-mediated demethylation may boost salt tolerance in tomato [80]. Furthermore, in *Pfaffia glomerata*, exposure to salt stress and 5-azacytidine treatment resulted in reduced growth and photosynthetic efficiency, altered metabolic activity, and a decrease in global DNA methylation levels as determined by HPLC. Both treatments also lowered the levels of 20-hydroxyecdysone (20-E), indicating that DNA methylation plays a regulatory role in the biosynthesis of this metabolite. Additionally, downregulation of a jasmonate biosynthesis-related protein highlighted the involvement of jasmonate signaling in 20-E production. These results highlight the interplay between epigenetic modifications and hormonal pathways in shaping salt tolerance and secondary metabolite biosynthesis in *P. glomerata* [81]. Tetraploid rice exhibits CHH hypomethylation near stress-responsive genes, especially those involved in jasmonic acid signaling, priming them for rapid activation under salt stress. Upon stress, transcriptome and methylome analyses show that gene expression induces CHH hypermethylation in nearby transposable elements, forming a feedback loop that enhances salinity adaptation. This consistent epigenetic pattern across tetraploid lines suggests a general mechanism of stress adaptation in polyploid plants [82]. In flax (*Linum usitatissimum*), global methylation decreased under salt stress, but was restored and enhanced upon application of epibrassinolide, a brassinosteroid hormone. Using MSAP analysis, genes affected by these changes were linked to key biological processes like vitamin B1 biosynthesis and protein regulation. This suggests that 24-epiBL seed priming helps plants adapt to salinity by modulating epigenetic DNA methylation and regulating stress-responsive pathways [83].

Salt-induced changes in DNA methylation often act in concert with histone modifications and chromatin remodeling, collectively influencing gene accessibility and transcriptional reprogramming essential for stress adaptation [16]. For instance, under salt stress, the *OsBZ8* locus in salt-tolerant Nonabokra rice displays reduced DNA methylation, enrichment of the active histone mark H3K4me3, and dynamic nucleosome positioning. In contrast, the salt-sensitive IR64 genotype exhibits higher levels of DNA methylation and accumulation of the repressive H3K27me3 histone mark, underscoring the pivotal role of epigenetic regulation in mediating their divergent stress responses [84]. Similarly, in poplar, overexpression of *PtRDM1* enhances DNA methylation primarily through chromatin remodeling associated with histone H3 modifications. This epigenetic reconfiguration significantly improves root regeneration under salt stress, suggesting that *PtRDM1* facilitates stress adaptation more effectively in chromatin-associated regions than in repetitive DNA sequences [85]. Beyond genic methylation, chromatin structure is also epigenetically influenced by salt. In rice, mild salt stress during germination causes decondensation of 45S rDNA chromatin and increased spacing between 5S rDNA loci, without affecting centromere positioning. 5-azacytidine treatment further enhanced 45S rDNA chromatin decondensation and centromere polarization, suggesting chromatin remodeling, influenced by DNA methylation changes, plays a role in rice salt stress adaptation [86,87].

Epigenetic regulation through both endogenous and exogenous factors, such as S-adenosylmethionine synthesis and foliar silicon application, plays a crucial role in modulating plant salt stress responses. I.n tomato, *SlSAMS1*, a gene encoding S-adenosylmethionine synthetase, the enzyme responsible for producing the methyl donor SAM required for DNA methyltransferase activity, plays a key role in epigenetic regulation under salt stress. Silencing *SlSAMS1* led to global DNA methylation loss and heightened salt sensitivity, while its overexpression enhanced salt tolerance by increasing CHG methylation within the gene body of *SlGI,* a circadian rhythm regulator, thereby upregulating its expression [88]. In maize, salt stress induced physiological and methylation changes, including elevated proline levels and altered DNA methylation patterns. However, foliar silicon (Si) application mitigated these effects in a dose-dependent manner by modulating DNA methylation, suggesting Si-induced epigenetic regulation as a mechanism of salt stress alleviation [89].

### 3.2. DNA Methylation During Drought or Water-Deficit Stress

Plants exhibit dynamic epigenetic modifications in response to environmental stimuli, with drought stress being one of the most critical abiotic factors affecting plant growth and crop productivity worldwide. Among these epigenetic modifications, DNA methylation plays a pivotal role in regulating gene expression and enhancing Drought or Water-deficit stress tolerance by orchestrating physiological, biochemical, and molecular responses.

Drought stress triggers the modulation of both DNA methyltransferases (5-mC MTases) and demethylases in various species, reflecting a highly dynamic regulatory system. For example, In *Ricinus communis*, most methyltransferases were downregulated except *RcMET1-2*, while demethylases like *RcDME*, *RcDML3*, and *RcROS* were significantly upregulated, suggesting a shift toward active demethylation to facilitate gene expression [90]. Another study in *Camellia sinensis* identified eight CsC5-MTase genes (classified into *CsMET*, *CsCMT*, *CsDRM*, and *CsDNMT2*) and four CsdMTase genes (grouped into *CsROS*, *CsDME*, and *CsDML*). CsC5-MTases showed overall downregulation, while demethylases (CsdMTases) increased under drought in *C. sinensis* [91]. In foxtail millet, drought triggered a showed a transient increase in most demethylase genes (*SiDML5*, *SiDML4*, *SiDML3a*, *SiDML3b*, *SiROS1b*, and *SiROS1d*), expression during the initial phase of drought, indicating time-dependent regulation, while *SiROS1a* consistently suppressed [71]. In eggplant, drought stress triggered coordinated regulation of DNA methylation genes, with six C5-MTases and five demethylases identified; most methyltransferases (*SmelMET1*, *CMT2*, *DRM2*, *DRM3*) and all demethylases were upregulated, while *CMT3a* remained unchanged and *CMT3b* was notably downregulated [70]. In apple, genome-wide analysis identified one *MdMET1*, two *CMT2*, three *CMT3*, one *DRM1*, and four *DRM2* genes. drought-induced transcriptional changes in DNA methylation machinery included peaks in most *MdDRM* and *MdCMT2* at 8 days, while *MdCMT3* expression rose early then declined and a steady increase in *MdDME* expression [92]. In citrus, three DEMETER-like DNA demethylases (CsDML1–3) were identified, showing low basal expression but responsiveness to drought, with CsDML1 and CsDML3 specifically regulated by deficit irrigation [93]. *HvDME* shows high expression during early seed development in barley, with distinct patterns between cultivars: in the large-seed variety Caresse, expression peaks at 5–10 days after flowering (DAF) before declining, while in the small-seed variety Ippolytos, it increases at 1–3 DAF and then remains stable. *HvDME* expression is strongly induced in drought-tolerant cultivars and varies during seed development, suggesting roles in both drought response and endosperm maturation [94,95]. CRISPR/Cas9-mediated knockout of *GmMET1* genes in soybean revealed that reduced cytosine methylation (mCG) leads to enhanced drought tolerance, with stress-responsive genes *GmNFYA5* and *GmNAC8* upregulated under decreased mCG levels. These findings highlight the functional importance of mCG in modulating stress responses and offer promising avenues for crop improvement [96].

Drought stress triggers a spectrum of DNA methylation changes in plants, ranging from global shifts to gene-specific modifications. These changes contribute to stress adaptation by modulating gene expression, enabling genotype- and tissue-specific responses, and potentially inducing heritable changes. For instance, *Populus trichocarpa* and *Morus alba* showed DNA methylation increases of 2.29% and 8.64%, respectively, under drought conditions, suggesting a protective role for hypermethylation [97]. Similarly, in wheat, methylation-sensitive amplified polymorphism (MSAP) analysis revealed that the drought-tolerant genotype C306 displayed more DNA demethylation than the sensitive HUW468 under drought, with significant polymorphisms across tissues and stages, highlighting genotype- and tissue-specific epigenetic responses [98]. At the gene-specific level, drought-induced demethylation at CNN sites in the Asr2 regulatory region in tomato roots suggests heritable changes promoting adaptive gene expression [99]. Concurrently, the *HvDME* gene, encoding a DNA glycosylase, is epigenetically regulated in barley under drought stress. Drought increases CG and non-CG methylation in its distal promoter while leaving the proximal region unmethylated. Presence of drought-responsive and transposable elements in its promoter supports its regulation through epigenetic mechanisms [94,95]. In rice, variable soil moisture levels induced differential methylation patterns, with higher methylation observed at 60% field capacity (severe drought stress) and lower levels at 100% field capacity compared to puddled conditions, suggesting methylation-based acclimation to water availability [100]. Further evidence of selective epigenetic control suggests that genes with CpG islands (e.g., *PP2C*, *PAL*) were downregulated under drought, while genes lacking CpG islands (e.g., *HSP70*, *RH25*) were upregulated, indicating that promoter architecture influences methylation-mediated expression regulation [100]. Methylation-Sensitive Amplified Polymorphism (MSAP) was used to assess DNA methylation changes in maize leaves under drought stress. Differential methylation of candidate genes, including lncRNAs, dehydration-induced protein 19, and nudix hydrolase 2, suggests their key regulatory roles in the drought response of maize inbred line W22 [101].

Drought-induced DNA methylation changes are highly specific to genotype and tissue, playing a crucial role in regulating gene expression and facilitating plant adaptation. For example, in faba bean, these changes were both genotype- and tissue-specific: the drought-tolerant genotype Bachar exhibited lower methylation levels in leaves and roots compared to the sensitive F177, along with higher expression of drought-related genes such as *LOX*, *CDPK*, *ABC*, and *GH*, suggesting a close association between demethylation and activation of stress-responsive genes activation [102]. Similarly, genome-wide bisulfite sequencing in *Populus* revealed that drought stress increased methylation predominantly in gene-flanking and repetitive regions. Importantly, methylation near transcription start sites tended to repress gene expression, whereas methylation within gene bodies and upstream regions was often linked to enhanced gene expression. Moreover, while most cis-splicing genes remained unmethylated, over 80% of trans-splicing genes exhibited methylation changes, highlighting the complexity of epigenetic regulation. Correspondingly, among transcription factors, 1156 showed decreased methylation and expression, while 690 showed increases, emphasizing their significant involvement in drought adaptation [103]. Supporting this, genes associated with differentially methylated regions (DMRs) in DK151 were linked to stress responses, indicating epigenetic control of drought tolerance through DNA methylation [104]. Furthermore, a separate study identified four methyltransferase genes exhibiting significantly higher expression in the drought-sensitive rice accession BX compared to the drought-tolerant LD10 during drought stress, suggesting that BX may undergo higher DNA methylation during drought. Global methylome analysis revealed similar overall methylation patterns (CG, CHG, CHH) in both accessions, with CG methylation being the most prevalent [105]. Notably, two drought-responsive genes, *CLT1* and *PSBP*, had elevated promoter methylation that decreased under drought, with their expression levels correlating to δ^13^C values and linked to DNA methylation changes, thus highlighting methylation’s regulatory role in their drought response. Notably, two drought-responsive genes in rice, *OsCLT1* (chloride transporter) and Os*PSBP* (photosystem II polypeptide), exhibited high promoter methylation under normal conditions, which decreased during drought, correlating with increased gene expression and δ^13^C value, showing a regulatory role for DNA methylation in drought adaptation [105]. In horse gram, methylation-sensitive amplified polymorphism analysis of drought-sensitive HPKC2 and drought-tolerant HPK4 showed higher methylation levels in HPKC2 (10.1%) compared to HPK4 (8.6%). Some altered methylation sites corresponded to genes such as *DRE binding factor* (*cbf1*), *POZ/BTB*, and *Ty1-copia retrotransposon*, which likely contribute to their contrasting drought tolerance and epigenetic profiles [106].

Drought stress in apple led to methylation changes in approximately 850 transcription factor (TF) genes. The sensitive cultivar ‘HC’ exhibited hypomethylation and upregulation of *MdOCP3*, a negative regulator of drought response, while the tolerant ‘QG’ showed no such change. Other transcription factors, including *DREB*, *WRKY*, *bHLH*, *HSP*, and *EIN3* families, also displayed cultivar-specific methylation and expression patterns, suggesting that epigenetic regulation of these TFs underlies the contrasting drought tolerance between ‘HC’ and ‘QG’ [92]. In sea buckthorn, a naturally drought-tolerant species, drought stress induced complex methylation dynamics, where 326 genes exhibited both differentially methylated regions (DMRs) and differential expression. A strong inverse relationship was observed, with hypermethylated genes being downregulated and hypomethylated genes upregulated. Notably, *VSR6* displayed promoter hypomethylation and increased expression, potentially influencing ABA signaling via cytosolic pH regulation. Other stress-related genes such as *PRAF1*, *LRK10*, *ZC3H18*, and *UPF2* also underwent methylation changes. Concurrently, the expression of methyltransferase genes *HrMET1* and *HrDRM1* was elevated, indicating activation of methylation machinery as part of the stress [107], In *Hibiscus cannabinus*, F1 hybrids showed enhanced drought tolerance associated with dynamic epigenetic modifications. MSAP analysis revealed that drought reduced methylation by 11.2% in the hybrids but increased it in both parents, with F1 plants exhibiting a 38% hypomethylation rate and more flexible methylation responses. Several key genes including *DnaJ*, *ERF5*, *ZIP2*, and *PATL3* showed altered methylation patterns potentially impacting their expression. Functional validation through gene silencing confirmed that *DnaJ* contributes to drought tolerance, underscoring the epigenetic basis of heterosis in drought response and providing useful targets for breeding [108]. Similarly, a study in wheat comparing the drought-tolerant ‘Bolani’ and the sensitive ‘Sistan’ revealed that ‘Bolani’ exhibited superior antioxidant capacity and water retention under drought, alongside greater CG and CHG demethylation. Gene-level analysis identified key demethylated genes such as *PEPC*, *BGlu*, *GT*, *GST*, and *LSD*, as well as methylated *ubiquitin E2* genes, all linked to improved drought tolerance, supporting the role of DNA methylation in cultivar-specific stress adaptation [109].

Small interfering RNAs (siRNAs) play a central role in RdDM pathway, which modulates drought tolerance through gene silencing. In maize, a MITE insertion in the *ZmNAC111* promoter recruited RdDM machinery, leading to gene silencing and reduced drought tolerance. Overexpressing *ZmNAC111* reversed the effect and improved water-use efficiency. This MITE insertion, likely post-domestication, contributed to drought adaptation in temperate maize [110]. In barley, drought stress induces hypermethylation of the HvCKX2.1 promoter via 24-nt hc-siRNAs, leading to repressed gene expression, educed cytokinin levels, and faster seedling emergence in the next generation, linking DNA methylation to hormonal regulation and transgenerational plasticity [111].

Accumulating evidence suggests that DNA methylation plays a vital role in establishing drought stress memory and enhancing adaptive responses in plants. In rice, the drought-tolerant line DK151 showed consistently higher methylation levels than sensitive variety IR64 in both leaves and roots. Under drought stress, DK151 exhibited approximately 12.1% genome-wide methylation changes, whereas the IR64 showed comparatively lower changes. Although, MSAP analysis suggested that many of these changes were reversible after recovery, some persisted in a tissue- and stage-specific manner, implying that DNA methylation contributes to stress memory and adaptation [112]. Moreover, drought stress induced cumulative and heritable methylation changes, with the drought-resistant variety Huhan-3 showing higher drought-induced (48.8% vs. 27.6%) and heritable (29.8% vs. 3.2%) differentially methylated loci, indicating greater stability and transgenerational transmission than the sensitive II-32B. These findings suggest that epigenetic inheritance via DNA methylation may contribute to enhanced drought resistance and offers a valuable target for rice breeding [113]. A genome-wide bisulfite sequencing study in rice revealed dynamic DNA methylation changes linked to drought memory that regulate gene expression and transposable elements during repeated drought cycles. Overall DNA methylation increased after initial drought and further rose following subsequent droughts, with CG and CHH contexts showing more responsiveness than CHG [114]. In *Medicago ruthenica*, DNA methylation may contribute to drought stress memory by promoting the transcription of key genes involved in abscisic acid (ABA) and proline biosynthesis. Specifically, the drought-related genes *ABA2* (involved in ABA biosynthesis) and *P5CS* (involved in proline synthesis) showed promoter and gene body hypomethylation and elevated expression in the D2 group compared to controls, with further upregulation following drought exposure. These memory-associated genes enable the plant to modulate its physiological responses more effectively during prolonged stress. Additional genes, including *PRP4*, *SAMDC*, *CYP707A2*, *MPK3*, *ERD15*, and *DRTH2*, also exhibited changes in methylation and expression patterns, reinforcing their roles in drought stress memory [115].

DNA methylation plays a crucial role in regulating gene expression under drought stress, affecting various physiological and metabolic pathways that contribute to plant adaptation across different species. For example, in citrus, *CsDML1* and *CsDML3* exhibited differential expression under deficit irrigation across fruit developmental stages, with their expression positively correlated with genes involved in carotenoid metabolism (*phytoene synthase*, *PSY*; *zeta-carotene desaturase*, *ZDS*) and citrate metabolism (*ATP citrate lyases*, *ACLs*), suggesting their involvement in drought adaptation [93]. In potato, transcriptome analysis revealed that DNA demethylation plays a key role in regulating *Glutathione S-transferase* (GST) genes under drought stress. Sixteen *StGST* genes responded to drought and demethylation, with expression patterns varying by drought tolerance. Overexpression of four *StGST* genes in tobacco enhanced drought resistance by boosting antioxidant and ROS-scavenging activity, indicating that GST-mediated stress tolerance likely regualted by DNA methylation [116]. In wheat, the glycolytic enzyme gene *TaGAPC1* (glyceraldehyde-3-phosphate dehydrogenase) exhibited stress-responsive expression regulated by promoter methylation. Under osmotic and salinity stress, the tolerant genotype Changwu134 showed contrasting promoter demethylation, while the sensitive genotype Zhengyin1 exhibited increased CHG and CHH methylation. Site-specific methylation changes in stress-related cis-elements highlight the functional relevance of promoter methylation under stress [117]. An apple gene *MdRFNR1* contributes to redox balance during drought and is regulated by a methylated miniature inverted-repeat transposable element (MITE-MdRF1) in its promoter. This methylated MITE is recognized by *MdSUVH1/3*, which recruit *MdDNAJ* proteins to activate one of the drought-responsive allele *MdRFNR1-1* [118]. A comprehensive analysis in rice identified 3064 memory transcripts with significant expression–methylation correlations, primarily regulated by CHH methylation. These transcripts were enriched in secondary metabolism, phenylpropanoid biosynthesis, and hormone signaling pathways, highlighting the critical role of DNA methylation in regulating metabolic pathways to establish drought memory in rice [119]. Interestingly, nitric oxide (NO) also modulates drought responses; in *Dendrobium huoshanense* seedlings subjected to simulated drought, treatment with 50 μM SNP (an NO donor) improved water retention, reduced damage, and enhanced antioxidant activity, while higher doses exacerbated stress. SNP treatment decreased global DNA methylation and promoted demethylation at key sites, suggesting that NO influences drought tolerance by modulating DNA methylation and mitigating oxidative damage [120].

### 3.3. DNA Methylation During Heat or High Temperature Stress

Heat or high temperature disrupts plant growth and development by causing structural and physiological damage. A rise of 10–15 °C above optimal levels defines heat stress, which can severely reduce productivity. To cope, plants employ signaling mechanisms to sense temperature changes and adjust cellular functions. They exhibit both basal thermotolerance (innate ability to survive sudden severe heat without prior exposure) and acquired thermotolerance (developed after mild heat exposure; involves stress memory and gene priming for improved resilience) [121]. Epigenetic mechanisms, especially DNA methylation, help regulate these heat tolerance responses.

High temperature disrupts plant cellular functions, making heat tolerance crucial for survival. Recent studies have shown that heat stress alters chromatin architecture by disrupting heterochromatin, increasing histone acetylation, and reducing DNA methylation. Specifically, two plant-specific histone deacetylases, HD2B and HD2C, are essential during heat stress for maintaining heterochromatin stability by promoting DNA methylation. They interact with ARGONAUTE4 to enhance its nuclear localization, linking histone deacetylation and DNA methylation in regulating chromatin under heat stress [122]. Heat stress triggers gene-specific active DNA demethylation during recovery in *Arabidopsis thaliana*, targeting genes involved in translation regulators and heat shock proteins, further emphasizing the heat stress adaptive role of methylation [123]. Similarly, the regulation of DNA methylation genes is temperature-sensitive. For instance, *ROS1*, a DNA demethylase, is downregulated, while *CMT3* is upregulated during Arabidopsis seed development under heat stress. In *ros1-4* mutants, defective germination and embryo lethality are linked to DNA methylation misregulation. Genes such as *ELF7*, *DEL2*, and *BRF3* were upregulated during germination in *ros1-4*, potentially impairing seed viability. These findings emphasize the essential role of ROS1-mediated demethylation in maintaining proper gene expression and seed viability under heat stress [124].

Accumulating studies highlight how heat-induced changes in DNA methylation influence various biological pathways, transcription factors, and stress response genes, revealing complex epigenetic regulation underlying heat tolerance. In maize (*Zea mays*), a genome-wide methylation analysis of seedling leaves under heat stress identified 325 differentially methylated genes (DMGs) that are potentially involved in heat adaptation. Functional annotation through Gene Ontology (GO) and KEGG pathway analyses showed that these DMGs participate in critical cellular processes such as DNA repair, RNA processing, starch biosynthesis, and key metabolic pathways including spliceosome assembly, RNA transport, and carbon metabolism. Importantly, many DMGs encode transcription factors belonging to families such as NAC, MYB, WRKY, and bZIP, all known for their roles in heat stress signaling. The study also revealed that nine spliceosome-related genes underwent demethylation coupled with increased expression, suggesting that heat stress may enhance alternative splicing through epigenetic regulation of splicing factors [125]. Similarly, in *Populus simonii*, temperature stress alters DNA methylation patterns that modulate microRNA (miRNA) expression. Using methylation-sensitive amplification polymorphisms, researchers identified 1066 differentially methylated sites, including seven miRNA genes. Subsequent qRT-PCR validation indicated that methylation changes influence miRNA expression levels, which in turn regulate target genes involved in lipid metabolism and oxidative stress responses. This epigenetic mechanism supports cellular survival and adaptation to temperature fluctuations in this woody species [126]. Expanding on these findings, cotton (*Gossypium hirsutum*) exhibits distinct methylation dynamics in response to high-temperature stress. Heat exposure decreased CG and CHG methylation levels while increasing CHH methylation, particularly in the D subgenome. Methylation density was found to be higher in chromosome centers compared to gene-rich regions. Integrated analyses of the methylome and transcriptome uncovered 5130 genes with coordinated epigenetic and expression changes, enriched in pathways related to hormone biosynthesis, thiamine, glutathione, and tyrosine metabolism—processes essential for stress adaptation. Notably, DNA methylation located in gene bodies or downstream regions generally promoted gene expression, whereas upstream methylation repressed it. These results emphasize the nuanced regulatory roles of DNA methylation in cotton’s heat tolerance and its promising applications for crop improvement [127]. In *Brassica rapa*, cold acclimation also induces DNA methylation remodeling that contributes to cross-adaptation to heat stress. Genome-wide methylation profiling identified 1562 DMGs, with about 60% showing changes in both promoter and gene body regions. Hypomethylation of promoters correlated strongly with increased expression of key genes such as *BramMDH1*, *BraKAT2*, *BraSHM4*, and *Bra4CL2*, which participate in pathways like GTPase activity, malate dehydrogenase function, the tricarboxylic acid (TCA) cycle, carbon fixation, and secondary metabolism. Functional validation demonstrated that *BramMDH1* enhances heat tolerance when expressed in *Arabidopsis*. However, chemical demethylation in *B. rapa* alone was insufficient to induce full cross-adaptation, suggesting that while promoter demethylation facilitates heat tolerance, it is not the sole determinant [128]. Short-term heat shock also causes DNA hypomethylation in *Brassica napus* microspores, predominantly affecting CG and CHG contexts. This methylation loss impacts both transposable elements and genes. Among differentially methylated genes (DRGs), *BnaA09g40710D*, an ELF3 homolog, was upregulated two-fold, linking DNA methylation status to temperature-responsive growth regulation. Another hypomethylated gene, *BnaC05g29060D* (similar to SCAMP5), is implicated in the heat stress response, highlighting the important role of methylation dynamics in microspore heat adaptation [129]. Moreover, heat stress induces genotype-specific methylation responses in *Brassica napus*. Heat-sensitive lines exhibit increased DNA methylation, whereas heat-tolerant lines show more demethylation. These epigenetic differences affect genes involved in calcium transport, mitochondrial function, and fucosyltransferase activity. Several heat-responsive genes, including calcium-transporting ATPase, protein kinase, and E1 C-terminal related 1, displayed expression changes associated with DNA methylation levels. This indicates that differential methylation plays a regulatory role in mediating genotype-specific heat stress adaptation [130].

*RDM16* is a heat stress responsive gene in *Arabidopsis thaliana* that enhances thermotolerance by regulating the splicing of heat shock transcription factors (HSFs). Loss of function mutants (*rdm16-1*, *rdm16-4*) are hypersensitive to heat, while complementation and overexpression of *RDM16* restore or enhance heat tolerance. *RDM16* promotes efficient splicing of key HSF pre-mRNAs, including *HSFA2* and *HSFA3*, and associates with these transcripts as well as U4, U6, and U3 snRNAs. It forms heat-induced condensates through arginine residues in its IDR1 domain, with its two splice variants, *RDM16 LONG* and *RDM16 SHORT*, working together to support thermotolerance. These findings reveal a novel mechanism linking mRNA splicing and phase separation to heat stress adaptation [131]. Heat stress also reduces rice seed size by disrupting endosperm development. Moderate heat accelerates endosperm cellularization, while severe heat blocks it. Heat represses the DNA methyltransferase *OsCMT3* and increases expression of *Fertilization-Independent Endosperm1* (*OsFIE1*), leading to smaller seeds. This suggests epigenetic regulation via *OsCMT3* repression and *OsFIE1* activation mediates heat-induced seed size reduction [132]. Chromomethylase 2 (CMT2) is a crucial epigenetic regulator involved in plant responses to heat stress and climate variability in *Arabidopsis thaliana*. It originated from an ancient duplication of CMT3 but lost its CHG methylation ability due to the loss of a critical arginine residue. However, a V1200R mutation restored both CHG and CHH methylation functions in mutants. Unlike the stable CMT3, CMT2 contains a flexible N-terminal domain that is essential for its function under heat stress but also renders it more sensitive and prone to degradation. Heat-induced degradation of CMT2 leads to a reduction in CHH methylation at heterochromatic transposable elements (TEs), potentially activating these elements. Over evolutionary timescales, such TE activation may increase genetic diversity and facilitate species adaptation to environmental stress [133]. A study further highlights the role of *CMT2* allelic variation in climate adaptation due to temperature. Climate-tolerant (plastic) alleles associated with *CMT2* and temperature seasonality have been identified in Arabidopsis populations. Specifically, a plastic allele at the *CMT2* locus was linked to altered CHH methylation patterns and enhanced adaptability to diverse climatic conditions. Correspondingly, *cmt2* mutants displayed greater tolerance to heat stress compared to wild-type plants, suggesting that genetic regulation of epigenetic variability contributes to phenotypic plasticity and environmental resilience [134]. The importance of *CMT2* in climate adaptation is reinforced by genome-wide studies linking it to environmental variation. Genomic data from diverse Arabidopsis accessions identified 16 climate-associated loci, including *CMT2*, that correlate with temperature seasonality. Accessions harboring the plastic *CMT2* allele exhibited modified CHH methylation profiles and increased thermotolerance, suggesting the epigenetic basis of adaptive responses to temperature fluctuations [134]. One such TE, ONSEN, a Ty1/copia-like retrotransposon, provides further insight into this dynamic regulatory landscape. ONSEN preferentially inserts into euchromatic regions and can modulate gene expression, such as by conferring ABA insensitivity. Although transposons are generally repressed by DNA methylation, CMT3 promotes ONSEN activation under heat stress by modulating H3K9me2 levels and supporting CMT2-mediated CHH methylation. In *cmt3* mutants, increased DNA methylation suppresses ONSEN activity, whereas *cmt2* mutants show reduced methylation and enhanced transposon activation [135].

Heat stress elicits epigenetic reprogramming in plants by modulating DNA methylation dynamics and activating components of the RdDM pathway. One key effect is the reduction in CHH methylation in transposon remnants in Arabidopsis, which leads to epigenetic misregulation. This disruption becomes more severe in RdDM-deficient mutants, as seen in *nrpd2*, where impaired RdDM pathway amplifies gene misregulation in adjacent regions. These findings highlight the importance of NRPD2-dependent RdDM in maintaining gene expression control and basal thermotolerance under heat stress [136]. Complementing this, heat stress also reshapes the expression profile of genes involved in the DNA methylation machinery. It upregulates *DRM2*, *NRPD1*, and *NRPE1*, implicating activation of the RdDM pathway via RNA polymerases IV and V (PolIV and PolV). This transcriptional activation appears to influence downstream demethylation pathways as well, as the DNA demethylase *ROS1* is notably downregulated in *nrpd1 nrpe1* mutants, suggesting it operates downstream of PolIV/PolV activity. Additionally, heat-induced promoter demethylation is linked to increased expression of stress-responsive genes such as Calmodulin-like 41 (*CML41*, At3g50770), although this correlation is not universal, as seen with At5g43260. These findings collectively demonstrate that PolIV/PolV not only mediate RdDM pathway but also orchestrate broader regulatory responses essential for heat stress adaptation [137].

Evidence highlights the role of N6-methyladenine (6mA) DNA methylation in regulating plant responses to heat stress (HS), particularly in rice. 6mA methylation is positively associated with HS tolerance, as global 6mA levels increase under heat stress conditions. Notably, the heat-tolerant cultivar 93-11 exhibits a stronger elevation in 6mA levels than the sensitive Nipponbare (Nip). In 93-11, this increases correlates with upregulation of *OsHSFA1*, a key heat shock transcription factor, and downregulation of *OsHSP70*, a negative regulator of *HSFA1*. These findings suggest that 6mA methylation orchestrates differential gene expression responses under heat stress, contributing to genotypic variation in thermotolerance [41].

### 3.4. DNA Methylation During Cold Stress

Cold stress, including chilling (0–18 °C) and freezing (below 0 °C), is a major abiotic factor that limits plant growth, productivity, and survival. It causes cellular damage, especially in hilly regions, but plants activate protective mechanisms. DNA methylation changes play a key role in regulating gene expression during these stress responses.

DNA methylation-related gene expression in plants undergoes dynamic changes in response to cold stress, highlighting epigenetic regulation as a key mechanism in stress adaptation. In foxtail millet, cold stress significantly altered the expression of all DNA demethylase genes. Notably, *SiDMLs* (*SiDML5*, *SiDML4*, *SiDML3a*, *SiDML3b*) and some *SiROS1s* (*SiROS1b*, *SiROS1d*) generally exhibited an initial increase followed by a decrease over a 24 h period, while *SiROS1a* displayed a unique fluctuating pattern with consistently lower expression compared to the control [71]. A similar regulatory complexity is observed in tea plants (*Camellia sinensis*), although studies on cold-responsive expression of DNA methyltransferase genes are limited. One study monitored transcript levels of *CsC5-MTase* and *CsdMTase* genes under cold stress over a 48 h period. The results revealed dynamic transcriptional changes, suggesting a potential role of DNA methylation in cold stress responses. Six *CsC5-MTase* genes (excluding *CsCMT1* and *CsCMT2*) showed marked downregulation after 12 h, with expression reaching their lowest levels at 48 h. In contrast, all *CsdMTase* genes showed a gradual increase in expression, peaking at 48 h [91]. Further supporting the role of DNA methylation in cold tolerance, studies in soybean demonstrated that *GmMET1* genes, which regulate cytosine methylation (mCG), influence both gene expression and developmental timing. Partial loss-of-function mutations in these genes altered flowering time and enhanced tolerance to cold and drought stress, underlining the critical role of mCG in coordinating development and environmental adaptability [96]. In the rubber tree cultivar Reyan 7-33-97, cold treatment activated key cold-responsive genes such as *HbICE1* and *HbCBF2*, as well as downstream cold-regulated (*COR*) genes and DNA methylation-related genes like *HbMET1*. Prolonged exposure to low temperatures resulted in promoter demethylation of *HbICE1*, *HbCBF2*, and *HbMET1*, leading to their enhanced expression. Additionally, genes associated with active demethylation (*HbDME*, *HbROS*, *HbDML*) were upregulated, although their own promoter regions did not exhibit methylation changes. A seasonal comparison across diverse cultivars revealed that the promoters of *HbICE1* and *HbMET1* were hypomethylated during winter and hypermethylated during summer, establishing a clear link between cold-induced demethylation and ambient temperature [138]. A similar epigenetic mechanism operates in sugar beet under cold stress. Cold exposure leads to global DNA hypomethylation, particularly in the CHH context, primarily due to the downregulation of DNA methylation-related genes, including *CMT2*, *SAM2*, and components of the RdDM pathway such as RNA polymerases IV/V, *CLSY1*, *RDRs*, and *DRB4*. Simultaneously, genes associated with active DNA demethylation, especially *ROS1*, along with *NPX1*, *PIE1*, and *SWC4*, are upregulated, whereas *DML2* is downregulated, indicating its comparatively minor role. This coordinated shift toward active demethylation, predominantly via the *ROS1* pathway, underpins the epigenetic remodeling necessary for transcriptional reprogramming and enhanced cold tolerance in sugar beet [139]. In cassava, cold stress induces tissue-specific methylation alterations, with petioles showing pronounced epigenetic remodeling. Differentially methylated regions (DMRs) were closely associated with the expression of cold-responsive transcription factors, including *ERF105*. Furthermore, miniature inverted-repeat transposable elements (MITEs), particularly those containing bHLH binding motifs, were highly sensitive to methylation changes, suggesting that dynamic regulation of these elements contributes to transcriptional plasticity during stress adaptation in tropical crops [140].

Cold stress induces extensive DNA methylation changes in various plant species, with demethylation occurring more frequently and playing a crucial role in stress adaptation. While DNA methylation is typically associated with the silencing of transposable elements, cold-induced demethylation can activate these elements, potentially contributing to the plant’s ability to respond rapidly to environmental stressors. In *Chorispora bungeana*, methylation-sensitive amplified fragment-length polymorphism analysis revealed that chilling and freezing stress elicited distinct epigenetic responses over a 24 h period. Genes involved in stress response, such as those encoding alcohol dehydrogenase, UDP-glucosyltransferase, and polygalacturonase-inhibiting protein, exhibited differential expression under the two cold stress conditions. These findings suggest that *C. bungeana* utilizes dynamic DNA methylation changes as a flexible mechanism for adapting to the variable cold conditions of alpine ecosystems [141]. Similar patterns have been observed in other species like upland cotton (*Gossypium hirsutum*), cold stress increases demethylation of both hemimethylated and fully methylated cytosines. Prolonged cold exposure results in reduced global methylation levels, which can be reversed upon return to normal temperatures. Corresponding expression changes were observed in stress-responsive genes: *GhCLSD*, *GhARK*, and *GhARM* were downregulated, while *GhTPS* was upregulated, in alignment with their respective methylation patterns. These results highlight the role of dynamic cytosine methylation in regulating gene expression under cold stress in cotton [142]. In maize seedlings, cold exposure triggers broad DNA methylation alterations, affecting approximately 32.6 to 34.8 percent of sites primarily through the demethylation of fully methylated fragments. Differentially methylated regions were associated with genes involved in hormone signaling, cold response, photosynthesis, and transposon activity. Notably, five homologous genes, including a receptor kinase, potassium channel, transposase, CBS domain protein, and TPR domain protein, showed increased expression under cold stress, suggesting that targeted demethylation serves as a rapid epigenetic mechanism supporting cold adaptation in maize [143]. Further insights into stress-responsive methylation come from studies on *msh1* mutants. These mutants exhibit enhanced tolerance to drought and salt stress but increased sensitivity to freezing. Under cold and low light conditions, *msh1* mutants display extensive non-CG methylation changes, specifically CHG methylation near centromeres and CHH methylation across the genome, at levels exceeding those in wild-type plants. However, these non-CG changes appear to have limited impact on the enhanced growth observed in *msh1*-derived progeny. Instead, heritable growth vigor is more closely associated with CG methylation changes, suggesting that while the *MSH1* methylome is more responsive to environmental stimuli, CG methylation plays a pivotal role in long-term phenotypic outcomes [144].

Cold stress in maize initiates a suite of epigenetic modifications along with the DNA methylation, which enhances the plant’s ability to respond to low temperatures. One prominent response involves the upregulation of histone deacetylases (HDACs), which leads to global deacetylation of histones H3 and H4. This chromatin remodeling promotes the transcriptional activation of key cold-responsive genes such as *ZmDREB1* and *ZmCOR413*. In parallel, cold exposure induces selective DNA demethylation and enhances chromatin accessibility near the ICE1 binding site of *ZmDREB1*, further facilitating its expression [145]. In addition to histone modifications, DNA methylation patterns in maize are also dynamically restructured under cold stress. Notably, demethylation predominantly occurs within nucleosome core regions, while linker DNA regions remain highly methylated. This results in a distinctive periodic methylation pattern aligned with nucleosome architecture. For example, the *ZmMI1* gene fragment, expressed exclusively under cold stress, displays alternating 45 bp hypermethylated and 145 bp hypomethylated segments. Such structured, non-random demethylation suggests a mechanism of broad transcriptional regulation in response to environmental cues, potentially contributing to heritable epigenetic memory without altering the underlying DNA sequence [146].

DNA methylation regulation, particularly through RdDM pathway and its antagonistic mechanisms, plays a critical role in plant responses to cold stress. In the RdDM pathway, small interfering RNAs (siRNAs) guide the methylation of DNA to silence transposons and certain genes, while the DNA demethylase ROS1 counteracts this process by actively removing methylation marks. This ROS1-mediated demethylation is essential for activating stress-responsive genes that contribute to abiotic stress adaptation. In *Arabidopsis thaliana*, exposure to cold induces ROS1-dependent demethylation in the promoter regions of *ACD6* (a gene involved in the salicylic acid pathway), *ACO3* (linked to ABA signaling), and *GSTF14* (a glutathione S-transferase gene), thereby upregulating their expression. Notably, ROS1 is particularly critical for the activation of *ACD6* and *GSTF14* under cold conditions [147]. Other regulatory components such as RNA-DIRECTED DNA METHYLATION 4 (RDM4) also contribute significantly to cold tolerance. RDM4, a protein associated with RNA polymerases Pol II and Pol V, modulates the *CBF* (C-repeat binding factor) cold-response pathway in *Arabidopsis*. Loss-of-function mutations in *RDM4* lead to reduced cold tolerance and downregulation of *CBF* genes and their downstream targets. In contrast, *RDM4* overexpression enhances expression of these genes and mitigates cold-induced damage. Importantly, RDM4 functions independently of the canonical RdDM pathway by promoting Pol II binding to the promoters of *CBF2* and *CBF3*, facilitating transcriptional activation during cold stress [148]. Furthermore, a paradigm-shifting study in *Arabidopsis thaliana* challenges the long-held view that the ICE1 transcription factor directly regulates *DREB1A/CBF3* expression during cold stress. It was discovered that the repression of *DREB1A* observed in the commonly used *ice1-1* mutant results not from the *ice1* mutation itself but from a transgene insertion that induces promoter hypermethylation via the RdDM pathway. Overexpression or deletion of *ICE1* does not affect *DREB1A* expression, suggesting ICE1 does not directly regulate *DREB1A* [149].

Recent studies have further expanded the role of non-coding RNAs in DNA methylation regulation under cold stress, emphasizing their importance in enhancing plant tolerance. In cassava, overexpression of the long non-coding RNA *CRIR1* significantly reduces global DNA methylation, leading to altered gene expression and improved cold tolerance. Comprehensive analyses of DNA methylation and transcriptome profiles revealed that *CRIR1*-induced hypomethylation is associated with the upregulation of specific transcription factors and genes involved in chlorophyll metabolism and photosynthesis. These findings offer new insights into the epigenetic mechanisms of cold stress adaptation and underscore the regulatory potential of lncRNAs in modulating DNA methylation [150]. Similarly, circular RNAs (circRNAs) have emerged as critical components in plant responses to chilling stress, particularly in economically important crops like tea (*Camellia sinensis*). This study found that circRNAs with longer flanking introns harbor more repetitive sequences and exhibit higher levels of DNA methylation, suggesting that repeat-mediated methylation facilitates circRNA biogenesis. These findings not only highlight the role of circRNAs in promoting cold tolerance but also offer promising directions for tea crop improvement through epigenetic and molecular breeding strategies [151].

Temperature-dependent regulation of transposon activity through DNA methylation has been well documented in various plant species. In *Antirrhinum majus*, the activity of the *Tam3* transposon is temperature sensitive, it is activated at 15 °C but suppressed at 25 °C. This temperature-dependent regulation is associated with increased DNA methylation at higher temperatures, which inhibits the binding of the Tam3 transposase (TPase). Notably, TPase can bind only during DNA replication at low temperatures, indicating that transposon-induced demethylation is a downstream consequence of its activation [152]. A similar link between DNA methylation and transposon regulation under cold stress is observed in maize. The *ZmMET1* DNA methyltransferase gene, typically expressed in dividing cells and associated with DNA replication, shows reduced expression in roots during cold exposure. This downregulation leads to selective DNA demethylation in the *Ac/Ds* transposon region, suggesting that *ZmMET1* plays a critical role in maintaining transposon methylation under stress conditions [153]. In cucumber, short-day, low-temperature conditions reshape the shoot apex methylome, causing widespread CHH demethylation around genes and transposable elements. These changes are accompanied by transcriptional reprogramming, including altered expression of ethylene-related genes such as *CsACO3* and an AP2/ERF transcription factor, which are implicated in temperature-dependent sex determination [154].

Epigenetic regulation of the ICE1–CBF signaling cascade plays a pivotal role in mediating cold tolerance across diverse plant species. In the invasive species *Ageratina adenophora* (crofton weed), cold tolerance has evolved during its northward expansion in China and is tightly associated with demethylation of the *ICE1* gene. This epigenetic modification leads to upregulation of the CBF pathway, enhancing cold adaptability. The observed epigenetic variation among geographically distinct populations suggests that demethylation-driven gene activation facilitates rapid evolutionary responses to colder environments [155]. *Arabidopsis thaliana*, natural variation in cold tolerance across geographic populations has also been linked to the methylation status of the *AtICE1* gene. Elevated methylation of *AtICE1* results in reduced expression of downstream CBF genes and diminished cold tolerance, whereas demethylation enhances freezing resistance, suggesting that epigenetic regulation of *ICE1* is a major determinant of regional cold adaptation in *Arabidopsis* [156]. A similar mechanism has been reported in *Oryza sativa* (cold-tolerant cultivar P427), where genome-wide DNA methylation analysis under chilling stress revealed dynamic epigenetic remodeling. Chilling led to the differential expression of 1654 stress-responsive genes, including members of key transcription factor families such as *MYB*, *AP2/EREBP*, *NAC*, and *WRKY*. Importantly, 51 genes exhibited concurrent changes in DNA methylation and transcriptional activity. Among them, *Os03g0610900*, a homolog of the *OST1* kinase involved in ABA signaling and the ICE-CBF-COR pathway, showed promoter demethylation and transcriptional activation, linking methylation status to ABA-mediated cold responses and enhanced tolerance [157].

DNA methylation plays a central role in regulating plant responses to cold stress by modulating gene expression involved in metabolic adjustments. It influences precise control of gene networks involved in lignification, aroma, pigmentation, metabolism, and antioxidant defense; these modifications offer promising avenues for enhancing cold tolerance and improving crop resilience in the face of climate variability. In tea (*Camellia sinensis*), linalool, a critical compound for floral aroma and defense, is epigenetically regulated under cold conditions. Two MYB paralog pairs activate linalool biosynthesis by forming MYC2–MYB complexes, whose activity is suppressed by JAZ proteins. Jasmonic acid (JA) enhances *CsMYB68* and *CsMYB147* expression, boosting linalool levels during tea processing. Cold stress further strengthens this pathway by activating JA signaling and promoting DNA demethylation, which increases MYC–MYB complex formation and enhances *CsMYB68* and *CsMYB147* expression. This connects linalool biosynthesis to both cold resistance and improved tea quality [158]. Similarly, in potato (*Solanum tuberosum*), the cold-responsive tricarboxylic acid cycle (TCAC) gene *StSUCLα1* undergoes DNA methylation changes at 11 intragenic cytosine sites, which are likely regulated by MET1. These methylation changes correlate with expression levels. Functional analysis through gene silencing in *Nicotiana benthamiana* reveals that suppressing its homolog increases cold sensitivity, indicating the methylation-dependent regulation of *StSUCLα1* in cold tolerance [159]. In another example, exogenous melatonin (MT) treatment helps alleviate cold-induced physiological deterioration in water bamboo shoots. MT enhances *ZlCDPK12* expression while repressing genes involved in the phenylpropanoid pathway (*ZlC4H*, *ZlCCR1*, *ZlCAD2*, *ZlPOD16*, and *ZlPAL1*), leading to reduced lignin deposition, browning, and weight loss. Concurrently, MT treatment maintains phenol and flavonoid content. These effects are associated with DNA methylation changes in transcription factors such as *ZlERF4*, *ZlbHLH49*, and *ZlMYC2.2*. These findings offer a foundation for improving cold tolerance in storage through metabolic pathway and DNA methylation [160]. In loquat (*Eriobotrya japonica*), cold stress-induced lignification is also regulated through DNA methylation. The gene *EjXND1* acts as a lignification repressor by inhibiting *EjHB1*-mediated activation of the lignin biosynthesis gene *EjPRX12*. DNA methylation in the *EjXND1* promoter, especially in regions where expression is negatively correlated, reduces the binding efficiency of transcriptional activators *EjHB3* and *EjMYB15*. This mechanism is modulated by modified Low Temperature Conditioning, which alleviates lignification through epigenetic control of *EjXND1* [161]. Supporting this mechanism, another study in red-fleshed loquat reveals that chilling stress reduces DNA methylation and upregulates *EjNAC5*, a key regulator of lignification. In contrast, white-fleshed loquat maintains higher *EjNAC5* methylation and lower expression, which prevents lignification. Overexpression of *EjNAC5* elevates lignin content and activates *Ej4CL1* and *EjPRX12* through interactions with *EjERF39* and *EjHB1*, unveiling DNA methylation-regulated modules of lignin biosynthesis [162]. Epigenetic regulation also influences pigmentation under cold stress. In blood orange, cold storage induces anthocyanin accumulation primarily in pigmented fruit regions by enhancing expression of genes such as *DFR* and *Ruby*. This is driven by promoter demethylation, likely due to cold-induced upregulation of the DNA demethylase *DML1*. Non-pigmented areas retain high methylation and show limited gene expression, highlighting an epigenetic basis for anthocyanin variegation [163]. Further evidence comes from *Fagopyrum tataricum* (Tartary buckwheat), where cold treatments, including cold memory and cold shock, result in global hypomethylation, especially in promoter and intergenic regions. This hypomethylation enhances the expression of cold-responsive metabolic genes such as *FtCuAO*, *FtRPB1*, and *FtDHE1*, which are involved in alkaloid, pyrimidine, and lysine pathways. These findings emphasize the importance of DNA methylation in regulating cold priming induced metabolic responses [164]. Recently, a study in winter rapeseed (*Brassica napus*) revealed that overexpression of the transcription factor *BnaHsfA2* enhances freezing tolerance by increasing antioxidant enzyme activity and reducing reactive oxygen species (ROS) and lipid peroxidation. Both transcriptomic and DNA methylation analyses show that *BnaHsfA2* is strongly upregulated under freezing stress at −4 °C for 12 to 24 h, confirming its pivotal role in epigenetically mediated cold stress adaptation [165].

Seasonal bud break in poplar is governed by dynamic changes in DNA methylation that align with cold stress signals. In spring, DNA methylation levels in the shoot apex tissue decrease, coinciding with the chilling-dependent induction of the DNA demethylase *PtaDML10*. Functional studies show that downregulation of *PtaDML8/10* delays bud break, and genome-wide analyses link DEMETER-like demethylation targets to the reactivation of growth, supporting the existence of an epigenetic mechanism that drives the dormancy-to-growth transition [166]. Complementary findings in chestnut and poplar further emphasize the role of DNA demethylation in seasonal responses. In both chestnut and its poplar counterpart *PtaDML6*, the DEMETER-like gene *CsDML* is triggered by cold and short days, two important environmental cues that cause bud dormancy. Overexpression of *CsDML* in hybrid poplar accelerates bud formation and increases flavonoid accumulation, suggesting that *CsDML* promotes early dormancy establishment to protect the shoot apical meristem during winter [167].

Emerging evidence highlights the critical role of DNA methylation in regulating vernalization-mediated flowering across various plant species. In sugar beet, DNA methylation significantly influences bolting during vernalization. High methylation levels in the shoot tip delay bolting, whereas low methylation alone is insufficient to induce it. Temperature treatments used for vernalization and devernalization alter both bolting behavior and methylation status, with lower methylation levels correlating with increased bolting rates. Moreover, different genotypes exhibit distinct methylation patterns and variable responses in key vernalization-related genes, such as *FLC* and *VIN3*, suggesting that epigenetic regulation of bolting could be harnessed for breeding cultivars with enhanced bolting tolerance [168]. Similarly, in *Brassica rapa*, vernalization leads to reduced DNA methylation of two casein kinase II subunits, *BrCKA2* and *BrCKB4*, which results in elevated gene expression and increased CK2 kinase activity. This modulation shortens the period of the circadian clock gene *BrCCA1*, thereby linking vernalization to photoperiodic regulation. Functional validation through gene silencing of *BrCKA2* and *BrCKB4* caused delayed flowering, reinforcing their role in the vernalization–photoperiod crosstalk [169]. In winter wheat, the central vernalization gene *VRN-A1* exhibits a distinct methylation profile in response to cold. Vernalization induces site-specific hypermethylation at non-CG sites within a transposable element-rich region of intron 1. This methylation pattern is mitotically stable but reset after reproductive development, suggesting that non-CG methylation in TE-rich regions contributes to the regulation of vernalization responses. These findings add an epigenetic layer to the control of *VRN1* expression during flowering induction [170].

Recent studies demonstrate that epigenetic and epitranscriptomic methylation modifications play crucial roles in plant cold adaptation. Mao et al. (2023) showed that low temperatures increase global levels of DNA N6-methyladenine (6mA) in Arabidopsis and rice, with these changes predominantly occurring within gene body regions. These 6mA modifications were closely associated with stress-responsive biological processes, and several key stress-related genes exhibited altered 6mA methylation following cold treatment. These findings revealed the important role of 6mA in cold adaptation and highlight its potential for improving stress tolerance in crops [171]. Complementing these DNA methylation insights, Ma et al. (2025) profiled RNA N6-methyladenosine (m^6^A) methylation in *Brassica rapa* under low-temperature stress, focusing on cold-tolerant varieties. Their results revealed significant alterations in m^6^A distribution, particularly an increase in m^6^A marks within the 5′ untranslated regions (5′UTRs) of mRNAs in highly cold-resistant lines. This modification correlated with enhanced expression of cold-responsive genes. Notably, the transcription factor *ZAT12* appears to act as a positive regulator via m^6^A-mediated expression, whereas *MYBC1* may serve a negative regulatory role. Key metabolic pathways influenced by m^6^A modifications include starch and sucrose metabolism as well as proline biosynthesis. Additionally, m^6^A methyltransferases and demethylases were differentially expressed under cold stress, emphasizing m^6^A’s vital role as an epitranscriptomic mechanism driving cold tolerance in winter *B. rapa* [172].

### 3.5. DNA Methylation During Heavy Metal Stress

In the modern era, anthropogenic activities have led to significant environmental contamination, with heavy metals emerging as a major class of persistent pollutants. Industrial emissions, mining, and agricultural runoff introduce heavy metals such as cadmium, lead, mercury, and arsenic into ecosystems, exerting deleterious effects on plant systems. These metals disrupt vital physiological processes, including metabolism, growth, and gene expression, often leading to oxidative stress and cellular damage [173]. Heavy metal exposure in rice induces heritable CHG hypomethylation and altered expression of methylation-related genes. These epigenetic changes persist in progeny, correlating with enhanced stress tolerance. The findings provide strong evidence for transgenerational epigenetic adaptation, where environmentally induced DNA methylation changes contribute to inheritable adaptive traits [174].

Among various heavy metals, cadmium (Cd) is particularly well-studied for its epigenetic impact on plants. Multiple studies have shown that Cd exposure alters DNA methylation patterns, often in a genotype- and tissue-specific manner, contributing to plant stress adaptation. Three rice genotypes (T-35, RZ-1, and RZ-2) were assessed for tolerance to Cd and other toxic elements (Cu and Cr). Miao et al. (2022) observed significant differences in gene expression, antioxidant enzyme activities, and DNA methylation patterns, particularly CHG hypomethylation, among genotypes, with T-35 showing stronger expression of heavy metal transporter genes. These findings suggest that DNA methylation underlies genotype-specific responses to toxic elements stress and also offer insights for future rice breeding strategies [175]. *OsMTP11*, a gene involved in metal tolerance, heavy metal homeostasis, and stress response, was found to be upregulated under Cd stress and other toxic elements such as Zn, Ni, and Mn exposure. Heavy metal stress led to decreased DNA methylation in the *OsMTP11* promoter, suggesting epigenetic activation of defense-related genes [176]. Similarly, the rice gene *OsGSTZ4*, a Zeta family glutathione-S-transferase, enhances Cd tolerance by promoting growth and reducing toxicity. Furthermore, Cd stress induces CHH hypermethylation at a nearby transposable element of the *OsGSTZ4* gene in rice, which is regulated by 24-nt siRNAs through the RdDM pathway. Promoter deletion confirmed the siRNA target site’s role in regulating *OsGSTZ4* expression, demonstrating a feedback mechanism that silences the transposable element and fine-tunes gene expression, highlighting an epigenetic strategy for rice adaptation to cadmium stress [177]. Feng et al. (2016) reported that cadmium exposure in rice induces widespread DNA methylation changes, particularly in genes related to stress response, metal transport, and transcription regulation. Most methylation-modified genes showed altered expression under Cd stress. Loss-of-function mutants for DNA methylation enzymes MET1 and DRM2 displayed generally lower transcript levels, with significant reduction in stress gene expression such as *OsIRO2*, *OsPR1b*, and *Os09g02214* in *drm2* mutants. Moreover, treatment with the DNA methylation inhibitor 5-azacytidine lowered methylation levels but enhanced rice seedling growth and cadmium accumulation, highlighting DNA methylation’s key role in regulating Cd response and tolerance [178].

Beyond rice, other plant species also show epigenetic regulation in response to cadmium stress. For instance, in *Nicotiana benthamiana*, Cd exposure alters DNA methylation and gene expression in four key genes (*NbMORC3*, *NbHGSNAT*, *NbMUT,* and *NbBG*). Silencing these genes increases Cd sensitivity, highlighting the critical role of DNA methylation in modulating stress-responsive gene networks [179]. Bai et al. (2025) found that in *Brassica rapa* ssp. *parachinensis*, microRNA miR9560, a 24-nt miRNA induced by Cd stress, regulates gene expression via the RdDM pathway. miR9560 modulates the expression of the Cd transporter gene *BrpHMA2* by inducing DNA methylation upstream of the gene, thereby limiting Cd uptake and enhancing tolerance [180]. Studies on *Amaranthus* species revealed that Cd exposure causes differential DNA methylation in roots and leaves, accompanied by altered expression of methyltransferase and demethylase genes. In root tissues, Cd stress led to the upregulation of chromomethylases (*AcCMT1*, *AcCMT2a*, *AcCMT3*), the de novo methyltransferase *AcDRM2a*, and the demethylase *AcDML2b*, indicating active methylation turnover. In leaves, Cd stress upregulated *AcDRM2a* and *AcDRM2b* but downregulated *AcCMT1* and *AcCMT3*, suggesting tissue- and gene-specific epigenetic responses to Cd toxicity. These findings highlight the role of epigenetic regulation in maintaining plant homeostasis under heavy metal stress [181]. In alfalfa, Cd stress downregulates eight DNA methyltransferases (*MsMET*, *MsCMT2*, *MsCMT3*, *MsDMS3*, *MsAGO4*, *MsDCL1*, *MsDRD1*, and *MsDDM1*) and reduces global DNA methylation, while DNA demethylase genes remain largely unchanged. Transgenic experiments demonstrated that overexpressing Cd-responsive genes (*MsNRAMP5* and *MsPCR2*) modified by 5mC significantly improved tolerance, reinforcing the functional role of DNA methylation and advancing stress-resilient alfalfa breeding [182]. Similarly, Luo et al. (2023) reported that cadmium (Cd) tolerance in kenaf hybrids is associated with global DNA hypomethylation. These hybrids exhibit enhanced Cd tolerance along with pronounced heterosis in growth and antioxidant activity. Analysis of 21 differentially methylated fragments identified Cd-responsive genes, such as *NPF2.7*, *NADP-ME*, and *NAC71*, that are regulated by cytosine methylation. Silencing the hypomethylated gene *NPF2.7* increased Cd sensitivity, suggesting that epigenetic deregulation may contribute to heterosis for metal tolerance [183]. In *Arabidopsis thaliana*, DNA methylation-deficient *ddc* mutants exhibited better growth under Cd stress. These mutants maintained higher levels of growth-promoting hormones (auxins, cytokinins, gibberellins) and lower levels of stress-related hormones (abscisic acid, jasmonic acid, and salicylic acid), indicating that hypomethylation confers hormonal and epigenetic flexibility for stress adaptation [184]. Interestingly, DNA hypermethylation also plays a protective and adaptive role. Cd pre-treatment reduces EMS-induced genotoxicity in *Allium cepa* and *Vicia faba* through hypermethylation and metabolic pathways. However, this adaptive response is abolished when DNA methylation is inhibited by 5-azacytidine, highlighting the essential role of methylation in maintaining genome stability under combined stress conditions [185].

Epigenetic mechanisms are also involved in plant responses to lead (Pb), which disrupts gene expression and methylation landscapes. In *Amaranthus*, Pb exposure significantly increased CG methylation maintenance and de novo methylation in roots by upregulating *AcMET1a* and *AcDRM2b*. In leaves, non-CG methylation was suppressed, with Pb strongly upregulating *AcDRM2a* and *AcDRM2b* while downregulating *AcCMT1*. These findings highlight tissue-specific methylation dynamics in response to Pb toxicity [181]. In maize, MeDIP-seq analysis revealed dynamic DNA methylation changes in roots under Pb stress. Differentially methylated genes due to Pb stress included key stress-related regulators such as MYB, bZIP, and F-box proteins, suggesting that epigenetic modulation of transcription factors plays a vital role in maize adaptation to Pb toxicity [186]. Interestingly, Tang et al. (2021) found that while Pb stress increases global methylation in radish, melatonin treatment induces DNA demethylation of heavy metal transporter genes and antioxidant genes. This reversal enhances detoxification pathways and improves Pb tolerance, illustrating the dynamic nature of methylation in stress response [187].

Chromium (Cr) stress disrupts growth and development and triggers epigenetic responses in plants, with DNA methylation and antioxidant gene regulation contributing to Cr tolerance. In kenaf, Tang et al. (2023) demonstrated that Cr stress reduces DNA methylation due to the downregulation of methyltransferase genes. However, exogenous application of glutathione (GSH) restores methylation levels, particularly in DNA repair and antioxidant-related genes, enhancing detoxification and tolerance [188]. Functional tests of the ROS-related gene *HcTrx* confirmed its role in enhancing antioxidant activity and Cr tolerance. This reveals that GSH aids Cr detoxification by modulating DNA methylation and activating antioxidant defense [188]. Guarino et al. (2024) showed that chromium (Cr) exposure in *Arabidopsis* alters gene expression and induces heritable DNA methylation changes. MSAP-Seq revealed altered methylation in stress-related, energy, and development genes in F1 plants. Reduced germination and growth in F2 seeds indicate that transgenerational epigenetic memory plays a key role in Cr stress adaptation, helping plants maintain defense mechanisms [189].

Aluminum (Al) stress primarily affects acidic soils, where its bioavailability increases. Epigenetic regulation under Al stress varies by genotype and tissue type. In triticale, Al-tolerant lines showed increased DNA methylation in roots, while non-tolerant lines displayed demethylation. Methylation in leaves remained unchanged, metAFLP and MSAP analyses detected Al-induced root-specific mutations and greater demethylation than de novo methylation at CCGG sites, suggesting a key role for DNA methylation in Al stress response and tolerance, suggesting that genotype- and root-specific methylation is crucial for Al tolerance [190]. In maize, Al stress caused DNA damage, changes in long terminal repeat (LTR) retrotransposon polymorphisms, and elevated DNA methylation, indicating extensive epigenetic remodeling of the genome. Al exposure reduced genomic stability and raised methylation levels. Polymorphisms in five barley-derived LTR retrotransposons suggest their mobilization and methylation changes contribute to Al stress defense [191]. Choi and Sano (2007) reported that Al stress in tobacco induced rapid demethylation in the coding region of the *NtGPDL* (glycerophosphodiesterase-like protein) gene, enhancing its transcriptional activation in response to metal and oxidative stresses. This suggests that gene body methylation dynamics can influence oxidative stress responses during Al stress [192]. In barley, Al tolerance is linked to a multiretrotransposon-like (MRL) insertion and promoter demethylation upstream of the *HvAACT1* gene, which controls citrate secretion for external Al detoxification from roots. Al-tolerant cultivars like FM404 exhibit low methylation (97.8% unmethylated) at this site, leading to higher *HvAACT1* expression, especially in root tips. In contrast, sensitive cultivars such as SV239 show high methylation levels. This epigenetic regulation of retrotransposon elements supports the local adaptation of barley to acidic soils [193].

Nickel (Ni) toxicity also elicits distinctive epigenetic responses in hyperaccumulator and non-accumulator species. In *Noccaea caerulescens*, a known Ni hyperaccumulator, exposure to Ni preserved nuclear integrity and increased DNA methylation compared to *Arabidopsis*. This response was correlated with upregulation of methylation-related genes like *MET1* and *DRM2*, supporting the role of methylation in genomic stability [194]. Similarly, Aina et al. (2004) reported dose-dependent DNA hypomethylation in *Trifolium repens* and *Cannabis sativa* under Ni^2+^, Cd^2+^, and Cr^6+^ stresses. Hemp showed higher baseline DNA methylation than clover. Using MSAP, metal-induced hypomethylation affected specific genomic sites, indicating that heavy metal-induced epigenetic changes are non-random and may contribute to adaptive responses [195].

In addition to the aforementioned metals, manganese (Mn), mercury (Hg), and Arsenic also trigger epigenetic alterations, affecting DNA methylation and gene expression. *Chenopodium ambrosioides*, a Mn hyperaccumulator, shows genotype-dependent DNA methylation patterns correlated with Mn accumulation and tolerance. Many methylated loci are linked to Mn accumulation, and key genes are regulated via methylation, underscoring DNA methylation’s role in Mn tolerance and the potential of epigenetic markers in breeding for phytoremediation traits [196]. Jing et al. (2022) showed that in pokeweed, Mn and Cd stress increase ROS levels and induce expression of DNA methylation-related genes (*CMTs*, *MET1s*, *glycosylases*). Inhibiting ROS generation reduced both methylation and gene expression, indicating that ROS acts upstream to mediate DNA methylation dynamics. These findings demonstrate that methylation changes under metal stress are ROS- and dose-dependent, highlighting epigenetic regulation as a key adaptive mechanism in pokeweed [197]. Regarding mercury, a Hg-tolerant rice mutant (*Osmet1-2*) was found to accumulate less Hg and exhibit increased survival. Promoter hypomethylation of 34 Hg resistance genes was observed, providing strong evidence of DNA methylation’s role in Hg detoxification and plant survival [198]. Arsenic stress affects plant epigenetics in a tissue- and age-dependent manner. In *Pteris cretica*, DNA methylation (5mC) was reduced mainly in older fronds under arsenic stress, despite higher arsenic accumulation in younger fronds. Methylation levels correlated positively with physiological performance and negatively with arsenic toxicity, suggesting age-specific epigenetic regulation influences arsenic accumulation and stress response [199].

## 4. Conclusions and Future Perspective

DNA methylation serves as a central epigenetic mechanism enabling plants to dynamically regulate gene expression in response to abiotic stresses. Through the addition or removal of methyl groups, primarily at cytosine or adenine residues, plants can silence or activate specific genes, thereby adjusting physiological and developmental processes to mitigate abiotic stress-induced damage. It has been demonstrated that DNA methylation patterns are profoundly influenced by salinity, drought, heat, cold, and heavy metal stress, resulting in hypermethylation or hypomethylation of key genomic regions. These changes influence the expression of genes involved in ion transport, antioxidative defense, hormonal regulation, and secondary metabolism. Plant responses to stress are often genotype- and tissue-specific, with certain methylation changes conferring epigenetic memory that enhances tolerance during subsequent exposures. This memory can persist through mitotic or even meiotic cell divisions, supporting the transgenerational inheritance of stress resilience. Furthermore, DNA methylation does not act in isolation but operates in concert with other epigenetic regulators, including histone modifications, chromatin remodeling, and non-coding RNAs. Hormonal cross-talk also modulates DNA methylation landscapes; for example, abscisic acid, gibberellins, jasmonates, and strigolactones provide signals to the methylation machinery to fine-tune transcriptional responses under abiotic stress conditions. Moreover, the recently recognized role of N6-methyladenine adds further complexity to gene expression regulation, with evidence suggesting it may positively influence transcription within gene bodies while exerting repressive effects in promoter regions. Despite these advances, several challenges remain. The causal relationships between specific methylation marks and phenotypic traits require further functional validation. While DNA methylation contributes significantly to stress tolerance, its application in epibreeding is constrained by the instability and limited heritability of many stress-induced marks. Overcoming this challenge will require precise strategies such as recurrent stress exposure, transgenerational studies, and comprehensive methylome tracking to identify reliable, heritable epialleles. Additionally, biotechnological tools necessary for targeted manipulation of methylation marks in crops are still in the early stages of development. Nevertheless, CRISPR-based epigenome editing and the identification of stable epialleles hold significant promise for developing stress-resilient plant varieties. Therefore, future research should aim to identify stable, heritable epialleles, advance CRISPR-based epigenome editing, and integrate epigenomic and phenotypic data to develop predictive models for stress-resilient crops. In conclusion, DNA methylation is a vital and versatile regulator of plant responses to environmental stresses. Its reversible and heritable nature provides a powerful adaptive mechanism that can be harnessed for crop improvement. Future research should integrate epigenomic data with transcriptomic and phenotypic analyses to construct predictive models for stress adaptation, guiding the next generation of sustainable and resilient agricultural systems.
